# Reconstruction of Rabbit Urethral Epithelium with Skin Keratinocytes

**Published:** 2015

**Authors:** O. S. Rogovaya, A. K. Fayzulin, A. V. Vasiliev, A. V. Kononov, V. V. Terskikh

**Affiliations:** N.K. Koltsov Institute of Developmental Biology, Russian Academy of Sciences, Vavilova Str., 26, Moscow, 119334, Russia; N.I. Pirogov Russian National Research Medical University, Ministry of Healthcare of the Russian Federation, Ostrovityanova Str., 1, Moscow, 117997, Russia; Morozov Children’s Clinical Hospital, Moscow Department of Health Care, 4th Dobryninsky Per., 1, Moscow, 119049, Russia; A.I. Evdokimov State University of Medicine and Dentistry, Ministry of Healthcare of the Russian Federation, Delegatskaya Str., 20/1, Moscow, 127473, Russia

**Keywords:** epidermal stem cells, keratinocytes, urothelium, cell plasticity, transdifferentiation, tissue engineering

## Abstract

We have investigated the living skin equivalent (LSE) as an alternative source of plastic material for closing full-thickness epithelial-stromal urethral injuries. The possibility of transdifferentiation of epidermal keratinocytes, a component of 3D tissue constructs, was investigated *in vivo *in a model of the recovery of urethral injuries in laboratory rabbits. Autologous grafting of LSE in de-epithelialized urethra showed that skin keratinocytes placed in a specific *in vivo *microenvironment can be incorporated into the damaged area and function as urothelium. The use of EGFP transfected keratinocytes allowed us to identify transplanted cells. The reconstructed urethral tubes did not develop strictures or fistulas at the site of the grafted LSE. Immunohistochemical studies of neo-urothelium revealed EGFP-positive cells expressing the urothelial markers K7 and UP3.

## INTRODUCTION

According to the current concept of the cellular mechanisms of regeneration, the nature and mechanisms of regeneration are defined by the types of tissue-specific stem and early progenitor cells involved in the process [1-3]. There is a significant amount of data demonstrating that under certain conditions tissue-specific stem cells can exhibit considerable phenotypic plasticity, which suggests a possibility of their transdifferentiation. Transdifferentiation has been successfully demonstrated in a number of models, including those for restoration of epithelial tissue in cornea, bladder, intestine, etc. [4, 5]. We believe that this phenomenon can be used in tissue engineering and cell technologies as an approach to create the cells and cell constructs required for the restoration of structures and/or functions of tissues and organs. Advances in this area of regenerative medicine will resolve the pressing issue of the shortage of a patient’s own tissues, which is implicated in a significant portion of reconstructive surgeries failures today [6, 7]. Another equally important aspect is the *in vitro *development of histotypic tissue constructs suitable for modeling morphogenetic processes, including those featuring tissue-specific stem cells [8-11].

In addition to direct incorporation into the damaged tissue structure, stem cells and cellular constructs can participate in repair and regeneration through induction [12, 13]. The effect may vary in its intensity and specificity. The induction can explain such phenomena as tissue recovery after transplantation of allogeneic tissue-engineering histotypic constructs containing stem/progenitor cells.

Taking this into account, the present study investigated the possibility of using the living skin equivalent (LSE) as an alternative source of plastic material for closing full-thickness epithelial-stromal urethral injuries. An *in vivo *model of the recovery of urethral injuries in laboratory rabbits was used to study the possibility of transdifferentiation of epidermal keratinocytes in 3D tissue constructs.

## MATERIALS AND METHODS

A total of 20 male chinchilla rabbits weighing no more than 2 kg were used in the experiments. All procedures were performed according to the rules established by the Bioethics Commission of the Institute of Developmental Biology, Russian Academy of Sciences. Animal experiments were conducted in accordance with Order № 267 of the Ministry of Healthcare of the Russian Federation issued June 19, 2003 “On the Approval of Laboratory Practice Rules.” The study group consisted of 14 animals; the control group, of six animals.

**Isolation and growth of rabbit keratinocytes**

All experimental animals had been assigned serial numbers prior to the start of the study. A tag with the number was attached to the cage in which the rabbit was kept for the duration of the study. The number was registered in the laboratory journal and subsequently assigned to the keratinocyte culture obtained from the rabbit.

At the first stage, skin samples were collected from the interior of a rabbit’s ear; the thickness of a biopsy slice was ca. 0.3 mm.

Immediately after the collection, the skin flaps were placed in a M199 medium with 4 mg/mL gentamicin. If necessary, the skin flaps were stored for 24 h at 4°C. All experiments were conducted under sterile conditions. Prior to storage, all skin samples were thoroughly washed with Hanks’ solution and placed in M199 or Eagle’s medium supplemented with antibiotics. Prior to cell isolation, the biopsy samples were washed with Hanks’ solution containing gentamicin (0.4 mg/mL) or 2000 U/mL penicillin and 1 mg/mL streptomycin. The skin flaps were cut into 3×10 mm strips, washed with PBS, and incubated in 0.125% dispase solution (Sigma) for 16–24 h at 4°C or in 2% dispase solution for 1 h at 37 °C. The epidermis was subsequently separated from the dermis along the basal layer with forceps. Pieces of the epidermis separated from the underlying dermis were washed with PBS and placed into PBS + 0.25% trypsin (1:1) solution. Following 10–15 min of incubation at 36°C, trypsin was inhibited by bovine or horse serum solution and a suspension of epidermal rabbit keratinocytes was obtained by pipetting. The suspension was then filtered through a 100 μm nylon mesh and centrifuged at 100*g* for 10 min. The supernatant was discarded, and the pellet was re-suspended in a keratinocyte growth medium. The keratinocyte suspension was seeded into plastic Costar cell culture flasks pre-coated with a collagen solution at a concentration of 200,000 cells/mL.

A liquid type 1 collagen (0.1 mg/mL) solution in 0.1% acetic acid was used to treat the working surface of the cell culture flasks. The procedure was performed as follows: 2–3 mL of the collagen solution was poured onto the bottom (25 cm^2^) of the flask and was kept at 37°C for 20 min. The collagen was then discarded, and the flask was thoroughly washed with Hanks’ solution supplemented with phenol red, until the disappearance of acid reaction.

For the first 3 days, rabbit keratinocytes were grown in a DMEM/F-12 (2:1) medium containing 10% fetal bovine serum (FBS), 5 μg/mL insulin (Sigma), 10-6 M isoproterenol (Sigma), and 5 μg/mL transferrin (Sigma). The cells were then switched to DMEM/F-12 medium (2:1) containing 5% FBS, 10 ng/mL epidermal growth factor (EGF), and other additives (see above) and cultivated in a CO_2_ incubator; the medium was replaced regularly.

**Destratification of keratinocyte culture layers**

Keratinocyte cultures were destratified after a multilayered sheet had been formed. The medium in the cell culture flasks was replaced with a Ca^2+^-free KBE medium, and the flasks were kept for 1–3 days until complete destratification of all cell layers, except for the basal one. After stripping, the keratinocyte cultures were switched to a normal growth medium and after 24 h were plated on the surface of the LSE. The keratinocytes were plated by removing them from the surface of the cell culture flasks with trypsin/EDTA (1:1) solution.

**Preparation of autologous rabbit keratinocytes and fibroblast-based LSE to be transplanted into urethral injury**

A Spongostan™ sponge package was opened under sterile conditions and a piece with a size and shape corresponding to those of a Petri dish was cut out with scissors and washed once with Hanks’ solution. Collagen gel containing postnatal human or rabbit fibroblasts at a concentration of 25–30 thousands cells per mL of the gel was prepared as described above and poured into the Petri dishes with Spongostan™ sponges. 1.5 mL of gel was poured into each Petri dish (O 3.5 cm).

Fibroblasts in collagen gel were cultivated on the sponge surface for 24 h in a CO_2_ incubator. This construct was then used as a connective tissue equivalent to prepare a LSE with fibroblasts in collagen gel and rabbit skin keratinocytes on its surface, according to the aforedescribed standard protocol. To observe the autologous grafting principle at all subsequent stages, the cell culture flasks with keratinocytes and LSE were marked with an adhesive label containing the number assigned to an experimental animal.

**Cell labeling with a tracer**

At the initial stage, the autologous keratinocytes were labeled with a 0.00001% solution of the DiI membrane tracer in a serum-free culture medium; the cells ready for transplantation were incubated in this medium for 1 h.

**Lentiviral transfection of the keratinocyte culture**

Transfection of cells with an enhanced green fluorescent protein (EGFP) was performed using the lentiviral construct (Evrogen; the virus is supplied in DMEM medium in the amount of 1.5×10^6^ virus particles/mL). The transfection was performed by introducing the amount recommended by the manufacturer (10 copies per cell).

For transfection, 1.5×105 keratinocytes were plated on a Petri dish (O 3.5 cm). After complete cell adhesion, the medium in the dish was replaced with the virus-containing DMEM medium. In order to increase the permeability of the cell membrane, polybrene at a concentration of 5 mg/mL was added simultaneously and the mixture was incubated for 24 h. After transfection, the cells were switched to the standard culture medium. EGFP expression was observed after 72 h.

## HISTOLOGICAL STUDIES

**Preparation of paraffin sections**

4% of paraformaldehyde in PBS (pH 7.4) was used for fixation. The tissue was fixed for 24 h and subsequently washed with PBS. The tissue was dehydrated through a series of alcohol baths according to the standard protocol. Xylene was used for further histological processing. The samples were then embedded in paraffin. The sections of paraffin blocks were prepared using a Carl Zeiss MICROM microtome. 5-μm-thick serial paraffin sections were prepared and transferred onto the glass. After deparaffinization, the sections were stained with hematoxylin and eosin.

**Preparation of cryosections**

Pieces larger than 0.5×1.0 cm were fixed for 1 h at room temperature in 4% paraformaldehyde solution, washed with PBS, and placed into 20% sucrose solution for infiltration for 8–12 h until full immersion. The materials thus prepared were frozen in nitrogen vapor and stored at –70°C. The 15- to 20-μm-thick sections were prepared using a Leica DM IL cryostat (Germany).

**Immunohistochemical studies of the preparations**

The expression of various proteins was measured using monoclonal antibodies to detect skin (K14) and urothelium (K7 and K18) keratins (NovoCastra), uroplakin 3(UP3) (UsBioLogical), and EGFP (Evrogen). The immunofluorescence method was used for antigen detection.

The following staining protocol was used for immunofluorescence studies: primary antibodies were diluted in a blocking solution (5% bovine serum albumin + 0.1% Triton X-100 in PBS) at the concentrations recommended by the antibody manufacturers and applied to the sections washed in 0.1% PBS. The samples were incubated for 12–16 h at 4°C. The material was subsequently washed 4–5 times with 0.1% PBS and incubated in Alexa-488 secondary antibody solution (Molecular probes) in PBS for 40 min in the dark at room temperature. To prepare temporary preparations, the samples were embedded in glycerol.

**Reconstruction of the experimental urethral injuries in rabbits**

**Fig. 1:**
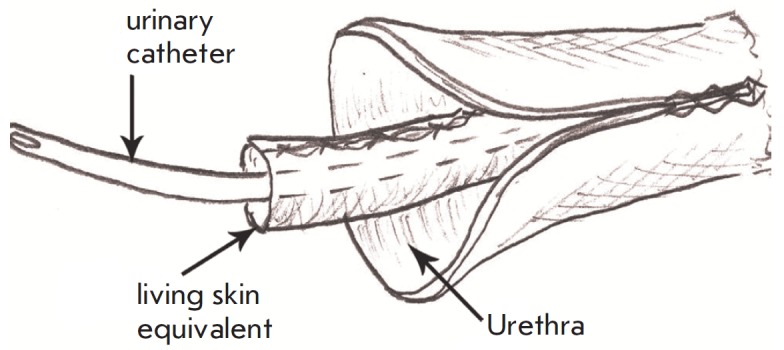
The scheme for LSE transplantation into de-epithelialized urethra

The surgery was performed under general anesthesia as described above. Additional novocaine blockade of the penis was performed simultaneously by introducing 0.5 mL of novocaine into the operating theater area. The surgery began with a circumferential incision around the glans penis followed by a longitudinal skin incision on the ventral side of the penis towards its root. Shaft skin was mobilized up to the root of the penis; the muscle was cut on the ventral side towards the urethral lumen. The epithelial lining of the urethra was then separated by forceps along the basal membrane for 1–1.5 cm up to the prostate section at the root of the rabbit’s penis. The rectangular collagen-based LSE, with a length equal to that of the urethral tube injury and a width equal to the circumference of the urethra being constructed, was stitched into a tube (*Fig. 1*). At the next stage, the proximal section of the urethra was anastomosed on a catheter to the remnants of the urothelium, stitching it to the tube, which was positioned between the distal de-epithelialized section of the urethra “end to end,” and to the top of the glans penis. In conclusion, the wound defect was sutured with local tissues. An 8 Ch. urethral catheter was sutured to the skin of the glans penis using atraumatic thread PDS 5/0. A compression bandage with glycerol was applied as the final stage. The urethral catheter was removed on day 7.

**Reagents and solutions used in the study**

M199x10 medium, DMEM/F-12 medium, Hanks’ solution, EDTA solution, PBS (PanEco LLC), fetal bovine serum (HyClon, USA); epidermal growth factor (Sigma), insulin (Sigma), isoproterenol (Sigma), hydrocortisone (Sigma), transferrin (PanEco LLC), HEPES (Sigma), antibiotics: gentamicin, penicillin, streptomycin (Ferein, Moscow), DiI Red membrane tracer (Sigma), lentiviral construct with *e-gfp *(Evrogen LLC, Moscow); trypsin solution (Biolot LLC, St. Petersburg), dispase (Sigma); plastic cell culture flasks (Costar, USA), Spongostan ™ gelatin medical sponge (Johnson & Johnson, USA). An inverted microscope Leica DM IL (Germany) and Kayensa conductive light microscope (Japan) were used for microscopy experiments.

Specific antibodies to keratins of epidermal basal layer cells K14 and differentiation markers of urothelium K7 and K18 (NovoCastra) and UP3 (UsBioLogical).

## RESULTS AND DISCUSSION

LSE were transplanted into de-epithelialized urethra of laboratory rabbits in order to study the plasticity of keratinocytes and analyze their behavior in the new microenvironment. One of the main prerequisites for potential incorporation of a graft into a damaged area is the absence of graft-versus-host reaction, which governed our choice of autologous skin cells for transplantation.

At the first stage a model of LSE transplantation into de-epithelialized rabbit urethra was developed (see the Materials and Methods section). The animals that had undergone surgery were capable of unassisted urination three weeks after the transplantation, whereby indicating the restoration of the urothelium function.

**Fig. 2:**
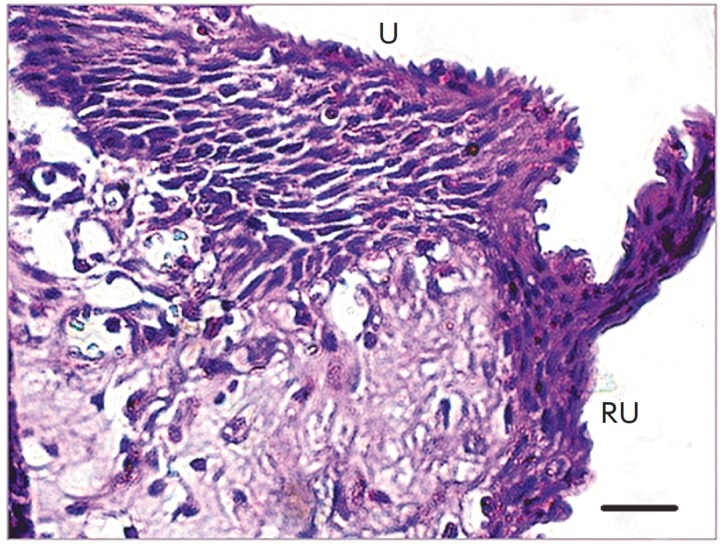
A section of the reconstructed portion of rabbit urethra 21 days after the transplantation. The site of the anastomosis between the host urethra (U, indicated by an arrow, upper part) and the restored urethra (RU). Stained with hematoxylin and eosin; scale bar length 20μm

The epithelium of the restored urethra differed significantly from the normal urothelium; its morphology matched that of flat multilayered epithelium (*Fig. 2*). This can be attributed to the choice of rabbit ear skin epithelium as a source of keratinocytes for construction of the LSE; nonetheless, the neoepithelium successfully functioned as the urothelium (unassisted urination, lack of fistulas). Since the objective of the first experiment was to develop a technology of graft preparation and surgery techniques, the behavior of the graft was monitored for 21 days.

In subsequent experiments, we used LSEs with labeled keratinocytes, which could be identified in the urethra. A total of 17 adult rabbits (6–12 months old) were used. Two groups of animals were used as a control: one group consisted of three animals with de-epithelialized urethra with no transplantation of the LSE, and the other one, of three animals that received a cellfree Spongostan™ gelatin sponge as a graft. The experimental group consisted of 11 animals grafted with LSE with the labeled cells. Within 2 weeks after surgery, all animals in the control groups developed complications, such as fistulas and a severe inflammatory response. After the catheter had been removed, four out of six control animals were incapable of unassisted urination. Therefore, the control group animals were sacrificed 2 weeks after the start of the experiment. All rabbits in the experimental group were capable of unassisted urination already on day 4–7 after the transplantation of the LSE. Neither urethral strictures nor narrowing of the tubes was observed.

The autologous cells were labeled with a DiI membrane tracer prior to transplantation. The resulting constructs were implanted into an experimental urethral injury site in seven rabbits. The outcome was evaluated 14, 30, 45 days, and 3 months after the implantation.

The animals’ urothelium had not been fully restored by day 14 after the LSE implantation. Small areas of epithelialization and cell clustering were detected, which contained DiI-labeled cells and cells positive for K14, a marker of skin epidermis basal keratinocytes. No cells positive for the urothelial markers K18 and K7 were observed.

Thirty days after transplantation, the histological sections revealed the presence of reconstituted epithelium, represented by one to three layers of cells. Virtually all its cells contained the membrane tracer and were positive for the urothelium differentiation marker K7 and basal epidermal keratinocyte marker K14. Keratin 18 was not expressed at this stage of urothelium restoration.

**Fig. 3:**
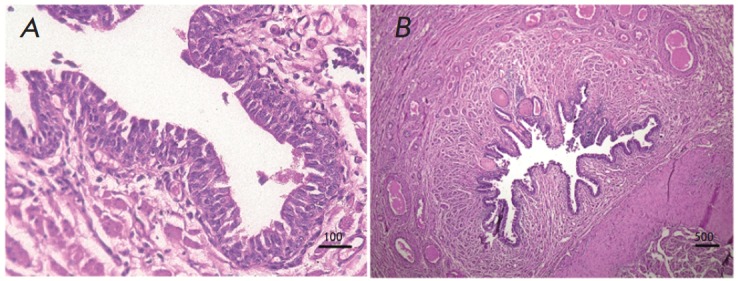
A section of the restored rabbit urethra 45 days after the transplantation. Stained with hematoxylin and eosinA section of the restored rabbit urethra 45 days after the transplantation. Stained with hematoxylin and eosin

The complete recovery of urothelium in rabbits, evident from the presence of a multilayer transitional epithelium, was observed 45 days after the transplantation (*Fig. 3*). The samples contained some labeled cells. Since the membrane tracer used for labeling in this experiment was diluted with every cell division, it had certain inherent limitations in terms of its detection in cells over time. By this point, all cells of the restored urothelium expressed K18 and K7. The presence of K14 in cells was weakly manifested. Therefore, the restored urethral epithelium matched the normal urothelium in rabbits in terms of keratin expression 45 days after the transplantation. The results obtained allowed us to conclude that the autologous skin keratinocytes grown in the LSE remain alive in rabbit urethra 1.5 months after the transplantation. The LSE transplantation allows one to restore the urothelium structure and function. In contrast to Atala [14], who used bladder biopsy samples as a source of cells, we used autologous epidermal keratinocytes, because the collection of autologous urinary tract epithelial cells significantly expands the operative field (biopsy of the bladder). This technology allowed us to resolve the issue of shortage of plastic material. Furthermore, it helps avoid the use of urethroplasty of skin containing hair follicles, which is often used by surgeons today.

The autologous epidermal cells used for transplantation can be incorporated into an injured area. The replacement of the missing tissue may be due to the compartment of the stem and transient cells that constitute the equivalent. Lehrer *et al*. [15] found that epidermal regeneration mainly occurs due to the basal layer stem cell compartment and transient cells. The rate of basal cell maturation and differentiation can be reduced. Li* et al. *[16] have shown that after the transplantation of keratinocytes grown in culture formations, the reconstruction of epidermis involves not only stem cells, but also some transient cells, including those that have already been committed to differentiation. Our findings and analysis of the published data suggest that when introduced into an urothelium microenvironment, stem cells and transient cells of the epidermis that are present in the LSE both perform urothelium functions and exhibit plasticity, thus acquiring the properties of urothelial cells.

In order to monitor the expansion of cell clones in the animal and to observe phenotypic plasticity under the influence of the microenvironment, we obtained cultures of epidermal skin keratinocytes from four rabbits and transfected them with a *e-GFP *lentiviral construct. A week after the transfection, 90% of the cells contained GFP and were strongly fluorescent in the green spectrum. The transfected keratinocytes were used to create LSE and were cultivated for one week.

The resulting graft with autologous cells was used to create the neourethra in rabbits as described above. Histological and immunohistochemical studies were performed 21, 45, and 90 days after transplantation. We used anti-EGFP antibodies to amplify the EGFP signal. In addition, the preparations were stained with antibodies against keratin 14 and 7, as well as the urothelial differentiation marker uroplakin III (UP3).

Twenty-one days after transplantation, a newly formed epithelium consisting of one to four cell layers was detected in the rabbit urethra. The epithelium was positive for EGFP in immunohistochemical experiments. This fact most likely indicated that we had detected the EGFP containing implanted skin keratinocytes in the urethral lumen. No UP3 was present in the restored urothelium at the time.

**Fig. 4:**
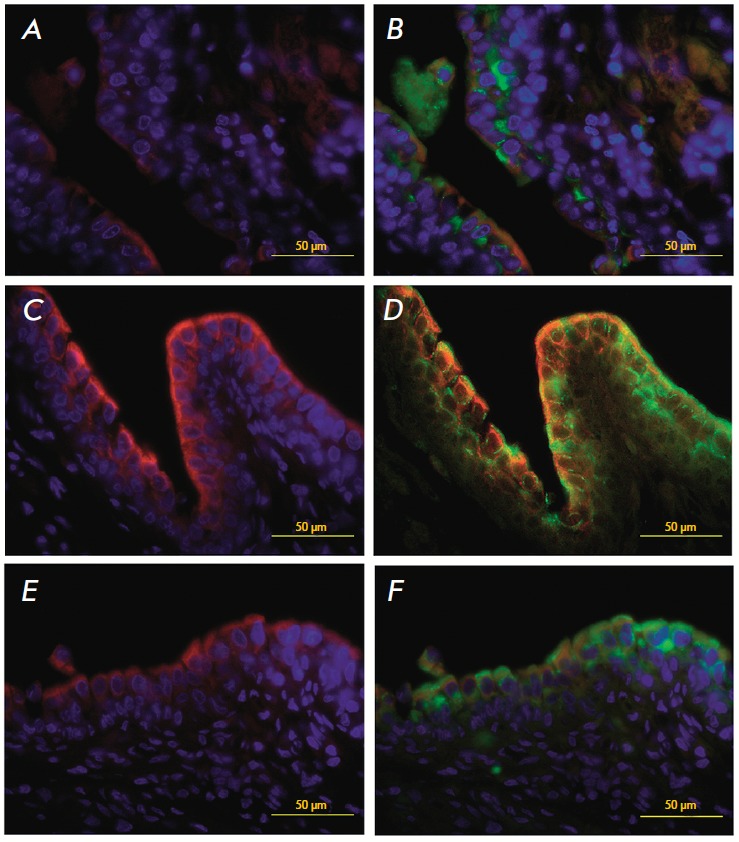
A cross-section of the restored rabbit urethra 45 days after the transplantation. A portion of the restored urothelium (immunofluorescent detection of urothelial markers). Colocalization of the EGFP genetic tag (B, D and F, green) and urothelial markers K14 (A and C, red), K7 (C and D, red), and UP3 (E and F, red). Nuclear staining with DAPI (blue)

Forty-five days after the transplantation, the urethral epithelium consisted of transitional epithelium which was three to seven layers thick. The EGFP-labeled cells were found in all layers of the urethral epithelium (*Fig. 4*), some of which also expressed keratin 14 (*Fig. 4 A,B*).

Other urothelial markers, K7 and UP3, have also been detected. High-magnification examination of the distribution of markers and EGFP expression in the neouretra strongly indicates colocalization of the green protein and the markers of differentiated urothelial cells (*Figs. 4C–F*).

**Fig. 5:**
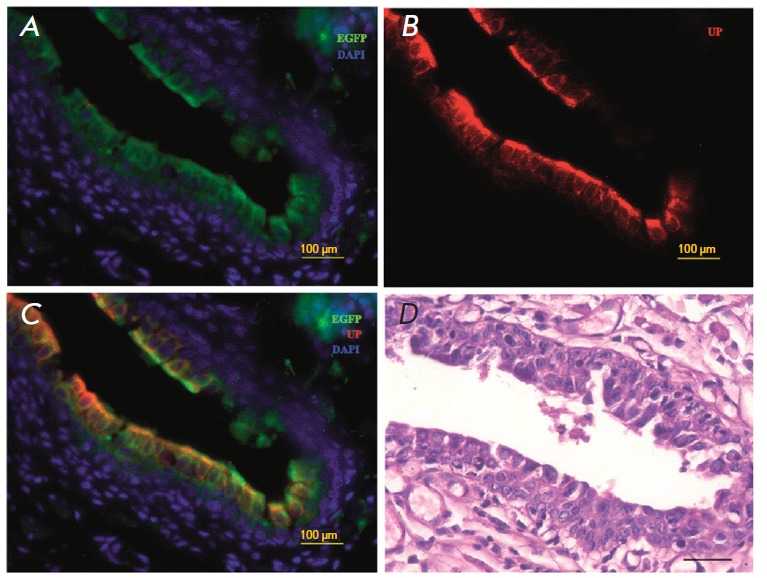
A cros-section of the reconstructed rabbit urethra 3 months after the transplantation. The restored urothelium (immunofluorescent detection of urothelial markers). Colocalization of the EGFP genetic tag (A and C, green) and urothelial marker UP3 (B and C, red). Nuclear staining with DAPI (blue). Stained with hematoxylin and eosin

**Fig. 6:**
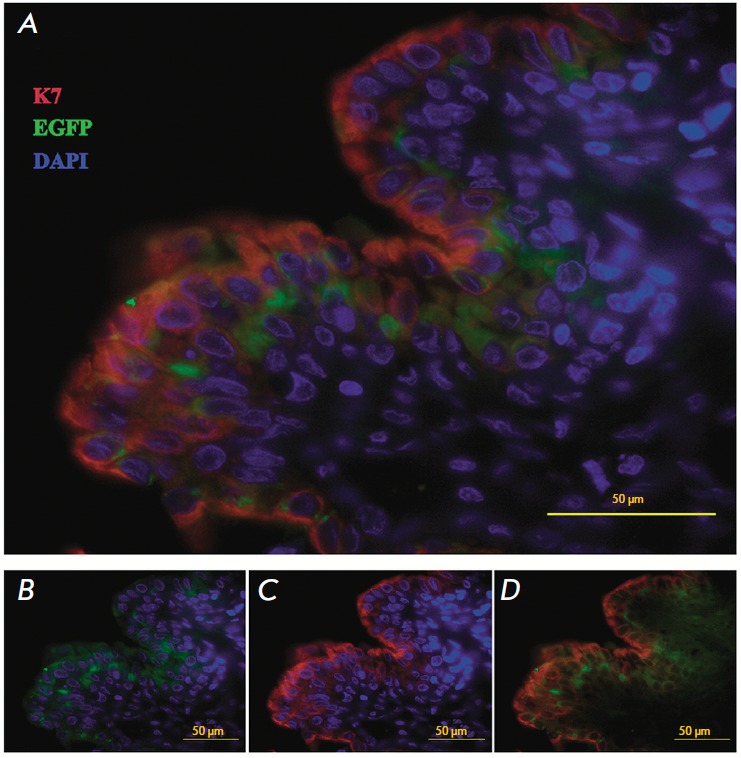
A cross-section of the reconstructed rabbit urethra 3 months after the transplantation. The restored urothelium (immunofluorescent detection of urothelial markers). Colocalization of the EGFP genetic tag (A, C and E, green) and urothelial marker K7 (A, C and D, red). Nuclear staining with DAPI (blue)

The restored urothelium preparations made three months after the transplantation had a fully formed urothelium with a normal structure and large proportion of top layer cells expressing UP3 (*Fig. 5*). EGFP-containing cells were also detected (*Fig. 5*, *Fig. 5*). The localization of label-containing cells can be explained by the fact that the regeneration rate in animals, in particular in rabbits, is rather high, and, therefore, the marginal migration of urothelial cells from the up per part of the urethra and proliferation of individual urothelial cells, which most likely remained in the site of the experimental injury, occurred in parallel with the widening of epithelialization islets of the transplanted keratinocytes. Identically to the previous experiments, all neourothelium layers expressed keratin 7 (*Fig. 6*).

The transplantation of autologous EGFP-containing rabbit skin keratinocytes into the urethral tube revealed that skin keratinocytes fully restore the urothelium within 3 months after their transplantation into the urethra. Urethral cells change their phenotype under the influence of the specific microenvironment, acquiring such features as expression of keratin 7 and UP3.

After being transplanted into an urothelial injury region, autologous skin keratinocytes incorporate into the urethra, restoring its integrity, and acquire the specific phenotypic traits of urothelial cells under the influence of the microenvironment.

The data suggest that adult skin keratinocytes grown *in vitro *and transplanted into the urethra can exhibit plasticity. This assumption is consistent with the data on the plasticity of adult stem/progenitor cells. In particular, it has been shown that epidermal keratinocytes exhibit plasticity under certain conditions. Under the influence of the seminal vesicles mesoderm of newborn rats, the differentiated cells of adult human urothelium start to express new non-specific markers of functional and morphological differentiation [17]. There is data showing that corneal cells can transdifferentiate into epidermal cells when exposed to fetal dermis signals [18]. Plasticity of epidermal keratinocytes has also been observed in experiments in cell transplantation into corneas. In [5], the authors investigated the phenotypic changes in genetically labeled cells and observed the transdifferentiation effect, which involved changes in the epidermal keratinocyte expression profile from K14 to K3/12 typical of the corneal epithelium.

The signals produced by the microenvironment determine the behavior and properties of stem cells. In particular, mesenchymal signals play a crucial role in maintaining their status. Ferraris *et al*. performed interspecies cross transplantations [19] and found that the signals from the embryonic mouse dermis can be recognized by adult rabbit corneal epithelium when it is transplanted under a renal capsule. Moreover, the corneal epithelium transdifferentiates into hair follicle- containing epidermis. The influence of urothelial- like cells on the phenotype of transplanted skin keratinocytes is also an important factor affecting the direction of their differentiation. In particular, this effect has been described *in vitro *for hair follicular keratinocyte cells grown in a medium conditioned by urothelial-like cells. As early as after 2 weeks of cultivation, skin cells cease to express K15 and begin expressing the urethral epithelium-specific keratins 7 and 18 [20].

These findings are both of theoretical and practical interest. The shortage of plastic material leads to a number of issues in reconstructive surgery of the genitourinary system. Atala [14] described a method for *de novo *creation of the urethra using bladder cells grown on a collagen substrate. The success rate of these surgeries suggests that tissue-engineering approaches to urethral reconstruction are very promising. However, this method has certain disadvantages, such as shortage of original plastic material and the invasive procedure of biopsy material collection from the bladder cavity. Many years of experience in using the skin (e.g. scrotum skin) as a readily available plastic material to create an artificial urethra have demonstrated that skin cells are can take root in an aggressive environment (adapting to the effect of the urine) and function as urothelium [21, 22]. However, the method can lead to complications such as hair growth in the urethral lumen during puberty. We propose a construction created from keratinocytes which have already passed the final step of cultivation and, therefore, no longer form hair follicles.

## CONCLUSIONS

We have developed an approach for urethral reconstruction using a collagen substrate and skin cells that have passed the final stage of cultivation. This urethra equivalent does not contain hair follicles and can be used in case of a shortage of plastic material. Autologous skin keratinocytes exhibit phenotypic and functional plasticity in a urothelial-specific microenvironment, acquiring the functions of urothelium.

Transplantation of the LSE with autologous skin keratinocytes into the site of a urethral tube epithelial injury in rabbits results in complete restoration of both the urothelium structure and the urethra function.

Transplantation of the LSE into the site of a urethral injury in rabbits leads to phenotypic changes in autologous epidermal keratinocytes, which acquire characteristics typical of urethral epithelium (K7 synthesis and UP3), whereby indicating the plasticity of adult epidermal stem cells.
